# The Ability of Exercise to Mitigate Caloric Restriction-Induced Bone Loss in Older Adults: A Structured Review of RCTs and Narrative Review of Exercise-Induced Changes in Bone Biomarkers

**DOI:** 10.3390/nu13041250

**Published:** 2021-04-10

**Authors:** Sarah J. Wherry, Ryan M. Miller, Sarah H. Jeong, Kristen M. Beavers

**Affiliations:** 1 Division of Geriatric Medicine, University of Colorado Anschutz Medical Campus, Aurora, CO 80045, USA; sarah.wherry@cuanschutz.edu; 2 VA Eastern Colorado Geriatric Research, Education, and Clinical Center (GRECC), Aurora, CO 80045, USA; 3 Department of Internal Medicine, Sections on Gerontology and Geriatric Medicine, Wake Forest School of Medicine, Winston-Salem, NC 27157, USA; millerr@wfu.edu; 4 Z. Smith Reynolds Library, Wake Forest University, Winston-Salem, NC 27109, USA; jeongsh@wfu.edu; 5 Department of Health and Exercise Science, Wake Forest University, Winston-Salem, NC 27109, USA

**Keywords:** caloric restriction, weight loss, bone loss, exercise, review, mechanisms

## Abstract

Despite the adverse metabolic and functional consequences of obesity, caloric restriction- (CR) induced weight loss is often contra-indicated in older adults with obesity due to the accompanying loss of areal bone mineral density (aBMD) and subsequent increased risk of fracture. Several studies show a positive effect of exercise on aBMD among weight-stable older adults; however, data on the ability of exercise to mitigate bone loss secondary to CR are surprisingly equivocal. The purpose of this review is to provide a focused update of the randomized controlled trial literature assessing the efficacy of exercise as a countermeasure to CR-induced bone loss among older adults. Secondarily, we present data demonstrating the occurrence of exercise-induced changes in bone biomarkers, offering insight into why exercise is not more effective than observed in mitigating CR-induced bone loss.

## 1. Introduction

Obesity is a prevalent, yet underappreciated, risk factor for fracture in older adults.

By 2030, an estimated 72 million Americans will be aged 65 and older [1], and half of them will be living with obesity [2]. Advanced age is a significant risk factor for osteoporosis [3,4], and the long-held view that obesity is osteoprotective has been recently challenged [5,6,7,8]. Indeed, data show that many, if not most, osteoporotic fractures occur in people who are overweight or obese [9], underscoring that the public health burden of osteoporotic fracture lies within this segment of the population. More recent evidence indicates that obesity adversely affects bone quality (i.e., bone structure and strength), which may explain the increased risk of fractures despite a predictably higher areal bone mineral density (aBMD) in the obese population [10,11,12]. Regardless of the underlying etiology, as osteoporotic fractures often significantly compromise both quality and expectancy of life [13,14,15,16,17,18], they are a hard clinical endpoint that older adults (and practitioners who manage their clinical care) strive to avoid.

Paradoxically, the treatment of obesity with caloric restriction further exacerbates the risk of osteoporotic fracture.

Observational data consistently link weight loss (WL) in older adults with loss of aBMD [19,20,21,22,23,24,25,26,27,28,29] and increased fracture risk [29,30,31,32,33,34], even among those who are overweight/obese and voluntarily losing weight [29]. Findings are echoed in randomized controlled trials (RCTs), where caloric restriction (CR) interventions of at least six months in duration are consistently associated with small, but significant, decreases in total hip aBMD (~0.010–0.015 g/cm^2^ and conferring a 10–15% increase in fracture risk) [24,35]. Results from the Look AHEAD study, which examined the effect of long-term CR (plus walking exercise) on fracture incidence in overweight and obese adults, confirm that 6–9% WL achieved and maintained over nearly a decade increases frailty fracture risk by 39% [31], with concomitant loss in aBMD [27,28]. Of additional concern, bone loss does not appear to be recovered with weight regain [36,37,38,39,40,41,42] and may even continue at an accelerated rate post-WL [41]. Thus, those who engage in repeated episodes of CR may further increase their fracture risk over time [24]. Collectively, data underscore recommendation to minimize bone loss during CR to reduce lifelong fracture risk in older adults undergoing obesity treatment [43].

Lifestyle-based strategies are currently recommended to mitigate CR-induced bone loss.

Lifestyle-based strategies, including dietary factors (such as calcium, vitamin D, and protein) [44,45,46] and exercise, have been proposed to mitigate CR-induced bone loss. Indeed, several studies show a positive effect of exercise on aBMD in weight-stable, older adults [47,48,49], with most recent recommendations indicating that exercise prescriptions designed to promote bone health should provide a multimodal approach consisting of progressive resistance exercise training (RT), bone-loading impact exercises, and balance training [50,51,52]. Specific exercise recommendations have been detailed previously by various working groups [50,51,52] and are summarized in Table 1. Briefly, recommendations include performing RT at least two days per week with moderate to heavy loads targeting major muscle groups, performing impact exercises (e.g., jumps or hops) on most days of the week (≥4 days), and achieving at least two to three hours of balance/mobility training per week. The latter recommendation targets fall risk, which is the primary driver of osteoporotic fracture [53]. Interestingly, aerobic exercise training (AT) has not been emphasized as part of these recommendations but is suggested to be included, given its additional cardiometabolic health benefits [54].

Literature examining the ability of exercise to mitigate CR-induced bone loss, however, is surprisingly equivocal [55,56,57,58,59,60,61,62,63,64,65,66,67]; we hypothesize it may be influenced by differing modes and intensities of the exercise prescriptions tested, outcome measures assessed, and/or potentially differential regulation of bone metabolism by CR and exercise. To better understand this equipoise, we conducted a structured literature review to identify and evaluate all published RCTs designed to evaluate the ability of exercise to mitigate CR-induced bone loss, specifically among older adults. Due to its robust change following CR [24] and clinical utility in predicting fracture [68,69], our primary outcome of focus was percent change in total hip aBMD [measured via dual-energy X-ray absorptiometry (DXA)], which is complemented by aBMD at the total body, femoral neck, and lumbar spine.

## 2. Structured Literature Review of Published RCTs

### 2.1. Search Strategy, Eligibility and Study Selection Criteria, and Data Extraction Process

**Figure 1:**
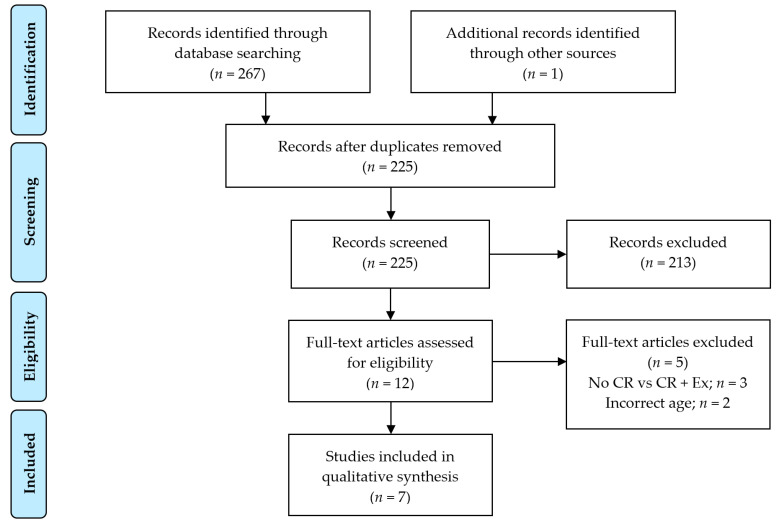
Flow diagram of included and excluded studies. Initial search conducted 20 December 2020; last search conducted 12 February 2021. Abbreviations: CR—caloric restriction; Ex—exercise.

To retrieve RCTs designed to evaluate the effect of exercise on CR-induced bone loss in older adults, systematic literature searches were devised and conducted in consultation with a science research librarian (SHJ). The Preferred Reporting Items for Systematic reviews and Meta-Analyses (PRISMA) statement was used for conducting this structured literature review (Figure 1). Search strategies used a combination of controlled vocabulary and keywords to achieve a balance of precision and recall (see Appendix A for a complete description of each search strategy), which were adapted and executed in four electronic bibliographic databases (MEDLINE in PubMed, Science Citation Index in Web of Science, Embase, and SportDiscus in EbscoHost) through 12 February 2021. Search terms included variations of the key concepts in the research question: “weight loss,” “exercise,” and “bone.” There were no restrictions on language nor publication dates, and animal studies were excluded by keyword search. Citations retrieved from electronic database searches and manual searching were imported into Endnote (*n* = 267, Version X9.3.3), and duplicates were removed (*n* = 43). One additional title not revealed during the database search was also added to the screening list [60]. Two reviewers (KMB and RMM) independently screened titles and abstracts (*n* = 225), ultimately including 12 full text articles being assessed for eligibility criteria based on conformation to design, age, and outcome criteria. Following review, five articles were eliminated (due to inappropriate study design or baseline age not reflective of an older population), leaving seven full-text articles retained in the qualitative analysis with relevant design characteristics [including sample size, gender breakdown, age, baseline body mass index (BMI), study duration, intervention descriptions, and achieved WL] extracted from each study. Change in DXA-acquired aBMD was also abstracted from each study, and, although total hip aBMD was the primary endpoint of interest (reported in *n* = 5 trials), we also abstracted data from trials reporting on change in total body (*n* = 5), femoral neck (*n* = 5), and lumbar spine (*n* = 7) aBMD.

### 2.2. Descriptive Characteristics of Included RCTs

Table 2 showcases descriptive characteristics of the seven included RCTs designed to assess the ability of exercise to mitigate CR-induced reductions in DXA-acquired aBMD among older adults [61,62,63,64,65,66,67]. As such, all trials were structured with participants randomized to CR alone versus CR plus an exercise condition. In brief, publication dates spanned nearly three decades, with older (age range: 54 to 70 years) women (*n* = 582, 75%) presenting with overweight or obesity (BMI range: 28 to 37 kg/m^2^) representing the majority of study participants. Three-to-four-month [61,66], 12-month [63,64], and 18-month [65,67] study duration endpoints were represented across the trials, with one study altering intervention delivery (from center-based exercise to home-based exercise) at six months and reporting on aBMD changes from baseline to 6 months and from 6 to 12 months separately [62]. Four trials included a multimodal exercise approach consisting of either RT plus AT [61,65] or RT plus AT, flexibility, and balance training [63,64]; one included separate RT and AT arms [67]; and one examined RT only [62]. Only three trials included a true control group [61,63,64].

Across trials, participants randomized to CR only lost a significant amount of body weight, which ranged from −3.3% to −12.2% of baseline values. Similar WL was achieved in most CR-plus-exercise conditions (WL range: −2.7% to −13.2%), with the lone exception of Beavers 2018 [67], where the addition of exercise (either AT or RT) augmented the degree of WL achieved via CR alone. In contrast, the majority of participants randomized to exercise-only or control conditions stayed weight-stable (−1.4% to +0.7%), except in the case of the Weiss 2017 [66], which utilized an exercise prescription specifically designed to induce significant WL. Across all studies, the magnitude of aBMD loss appears to parallel the quantity of WL, with approximately a 10% reduction in body weight corresponding to 1–3% of aBMD loss at axial skeletal sites. Altogether inconsistent observations regarding the ability of exercise to mitigate CR-induced bone loss were reported across studies (and skeletal sites), with three studies reporting some benefit [62,63,64], and four showing a null [65,66,67] (or even negative [61]) effect of exercise on aBMD. Trial-specific findings, organized by study duration and publication date, are presented below.

### 2.3. Short-Term RCT Results

Two RCTs, published nearly 25 years apart and lasting three to four months in duration, examined the short-term effects of exercise on CR-induced bone loss among older adults [61,66]. The first trial was published by Svendsen et al. in 1993 and reported on 121 older (53.8 ± 2.5 years) women randomized to control (maintenance of normal lifestyle; *n* = 21), CR-alone (targeting ~1000 kcals/day; *n* = 51), and CR-plus-exercise (*n* = 49) conditions [61]. Exercise was supervised, with sessions completed three days per week, consisting of both AT and RT, and gradually increasing in intensity and duration (from 1 to 1.5 h per session) throughout the three-month period. Specific exercise intensities were prescribed at 70% of maximum oxygen uptake and approximately 65% one-repetition maximum (1-RM) for AT and RT, respectively. Participants completing CR alone and CR plus exercise achieved similar WL (−12.2% and −13.2%, respectively), which was significantly greater than that of the control group (+0.7%). Comparable declines in total body aBMD were observed in the CR-only and CR-plus-exercise arms (both −1.9%), which slightly (though not significantly) exceeded that of the control group (−1.2%). Lumbar spine aBMD, however, significantly decreased in CR-plus-exercise (−2.4%) versus CR-alone and control conditions (−1.6% and −0.4%, respectively).

More recently, Weiss et al. randomized 52 older adults (57 ± 5 years, 75% women) to a four-month trial consisting of CR alone (20% energy deficit for 12–14 weeks; *n* = 17), AT (~60 min of moderate to vigorous AT per day; *n* = 16), or CR plus AT (*n* = 19) [66]. Of note, and in contrast to all other included trials, each intervention strategy was designed to decrease body weight similarly by 6−8% (actual achieved WL ranged from 6.9 to 7.4%, with no difference between treatment groups), and, at study close (3.5 months), no change in aBMD was observed at any skeletal site assessed. While the lack of change could certainly be influenced by the short study duration, additional explanations include that the degree of achieved WL did not exceed a critical threshold necessary to induced bone loss and/or that exercise-induced WL does not affect bone in the same way as CR-induced WL (as previously shown [58]).

In summary, and although limited, data from short-term studies do not support the notion that exercise can mitigate CR-induced bone loss and may even suggest skeletal harm with combined interventions. However, as bone remodeling typically requires a minimum of 4 to 6 months and can continue for at least 12 months [70], we focused on weight observations from the five trials lasting at least six months in duration when drawing conclusions from the available literature.

### 2.4. Long-Term RCT Results

The first published long-term study was conducted by Daly in 2005 and examined 6- and 12-month changes in bone outcomes among adults with type 2 diabetes randomized to CR alone (*n* = 13) or CR plus RT (*n* = 16) [62]. During the first six months, CR was individually prescribed to induce 0.25 kg WL per week, while the CR-plus-RT arm likewise received the same CR prescription and performed supervised RT three days per week at 75 to 85% 1-RM on a combination of upper and lower body exercise machines. From 6 to 12 months, CR-plus-RT participants transitioned from a center-based to a home-based exercise regimen (including use of dumbbells and ankle weights three days/week, with the goal of exercising at 60–80% 1-RM intensity) and returned to free-living feeding conditions. Both arms achieved significant, and similar, WL during the first six months (~−3%), and no differences in femoral neck or lumbar spine aBMD change were observed during the first six months of intervention. However, total body aBMD declined significantly more in the CR-alone group versus the CR-plus-RT one (−0.9% versus −0.3%, respectively). Interestingly, from 6 to 12 months, both groups significantly increased body weight (~ + 2%), yet aBMD did not rebound, with estimates revealing continued (albeit non-significant) losses across all skeletal sites.

The remaining trials include timepoints of 12 months or longer [63,64,65,67], with two trials reporting observations from the same cohort [63,64]. Specifically, both Shah et al. [64] and Villareal et al. [63] reported on data from the same study sample of 107 older adults (>65 years of age) living with obesity, who were randomized to 12 months of control (prohibited to complete outside exercise; *n* = 27), CR alone (caloric deficit of 500–750 kcals/day; *n* = 26), multimodal exercise (three days per week lasting ~90 min and consisting of AT, RT, flexibility and mobility exercises; *n* = 26), or CR-plus-multimodal-exercise (*n* = 28) conditions. Skeletal endpoints presented in Villareal et al. [63] include change in total hip, total body, and lumbar spine aBMD, while Shah et al. [64] presents change in similar sites, in addition to trochanter and total hip aBMD and biomarkers of bone turnover and metabolism. Participants randomized to the control and exercise-only groups displayed significantly less WL (~−1.0%) compared to CR-alone and CR-plus-exercise groups (−10% and −9%, respectively). No group differences were noted for total body or lumbar spine aBMD, but significant results were observed at the total hip and femoral neck. Specifically, for total hip aBMD, participants randomized to either CR condition saw significant reductions from baseline; however, loss was attenuated in the group receiving concurrent exercise treatment (−1.1% versus −2.6%). Similar findings were noted at the femoral neck, with CR-plus-exercise participants also experiencing attenuated loss compared to CR-alone participants (−0.9% versus −2.1%).

Finally, two studies by Beavers et al. examined the effects of CR and exercise on 18-month bone endpoints among older adults living with specific chronic conditions [65,67]. In the first publication, the independent and combined effects of CR and exercise on aBMD were examined in overweight and obese older adults with osteoarthritis [65]. Intervention arms consisted of multimodal exercise (three days per week of AT for 15 min, RT for 20 min, AT for 15 min; *n* = 95); CR alone (caloric deficit of ~800–1000 kcals/day; *n* = 88); and CR plus multimodal exercise (*n* = 101). The AT sessions were completed at 50–75% heart rate reserve, whereas RT targeted lower-body muscle groups where 1–2 sets of 10–12 repetitions were performed [71]. All groups displayed significant WL from baseline; however, the exercise-alone group displayed modest losses (−1.4%) as compared to the CR-alone and CR-plus-exercise groups (~−10%). No differences were noted across groups for change in lumbar spine aBMD; however, participants in the exercise-only group lost significantly less total hip (−0.2%) and femoral neck (−0.2%) aBMD compared to those in both CR-alone and CR-plus-exercise arms. Specifically, both CR groups experienced a −1.6 to −1.7% loss in femoral neck aBMD and a −2.0 to −2.4% loss in total hip aBMD. Although exercise was not able to mitigate CR-induced aBMD loss at the hip region, interestingly, the authors note that the clinical classification of osteopenia or osteoporosis was unchanged from baseline.

In the more recent study from this group, Beavers 2018 investigated bone outcomes in older adults with cardiovascular disease or metabolic syndrome who were randomized to one of the following 18-month prescriptions: (1) CR alone (caloric deficit of ~330 kcals/day; *n* = 60); (2) CR plus AT (45 min four days per week; *n* = 67), or (3) CR plus RT (upper and lower body machine-based exercise four days per week; *n* = 60) [67]. The authors set out to determine the effect of exercise modality on WL-associated bone loss, and this was the only identified trial which assessed quantitative computed tomography (QCT)-derived changes in volumetric (v)BMD, cortical thickness, and strength (albeit in a subset of participants, *n* = 55) in addition to DXA-derived aBMD. The CR intervention was designed to promote intensive WL during the first six months of the study, followed by a transition to WL maintenance for the duration of the trial. Exercise prescriptions were similar in frequency (4 d/week), intensity (rating of perceived exertion, AT: 12–14; RT: 15–18), and duration (45 min/day). Additionally, legacy effects of the intervention were examined at 30 months follow-up (after one year of free-living conditions). At 18 months, although CR-alone participants lost significantly less body weight than those in the CR-plus-AT or RT groups (−5.9% versus ~−10%), no differences were observed across groups regarding change in total hip, femoral neck, or lumbar spine aBMD. However, a significant loss from baseline evaluated between −2.3 and −3.7% in hip aBMD was observed in all groups. Interestingly, after adjustment for the degree of WL and followed out to 30 months, secondary analyses revealed that CR plus RT modestly mitigated total hip (−1.8%) and femoral neck (<−0.1%) aBMD loss compared to CR alone (−2.5% and −1.2%, respectively), with the magnitude of 18-month QCT-acquired hip vBMD treatment effects corroborating the DXA findings [and showing the decline in total hip vBMD was nearly halved by CR plus RT (~−5%) compared to CR alone and CR plus AT (both ~−9%)].

### 2.5. Summary

Taken together, several conclusions can be drawn from the available data. First, the literature base is small, with lack of consensus regarding the ability of exercise to mitigate CR-induced bone loss. Indeed, the strongest evidence in support of a beneficial exercise effect comes from two studies published from the same cohort, which implemented an aggressive (and perhaps efficacious, but not effective) multimodal exercise prescription to mitigate bone loss, with most pronounced effects observed at the hip region. Subsequent trials published by this group [72,73] support the general finding that RT offers superior skeletal protection (as compared to AT), although the ability of exercise to confer skeletal benefit appears modest at best. This underwhelming response may stem from a lack of compliance [74] or a blunted anabolic response in this age group [75]. It could be, however, that exercise is more catabolic to bone than generally recognized, which may diminish the mechanical loading benefit of exercise [76], particularly in the context of CR. The aim of the next section of this review is to highlight emerging research focused on exercise-induced bone resorption as an underappreciated catabolic consequence of exercise for bone.

## 3. The Effect of Exercise on Markers of Bone Resorption and Formation

One of the primary mechanisms by which exercise and CR are believed to exert similar, yet opposing, effects on bone is through mechanical (un)loading [77,78]. Coined as the “mechanostat” over three decades ago [79], this theory posits that bone adapts its morphology and strength in response to loading conditions as a part of a continuous negative feedback loop. This mechanical signal is then detected by cells within the bone, presumably as fluid sheer stress sensed within the osteocyte, which in turn generates secondary biologic signals governing the modeling and/or remodeling response [80]. In support of this general premise, and as observed in many of the RCTs included in this review, the magnitude of CR-induced aBMD loss often associates with the magnitude of WL, a finding which extends to the surgical WL literature [81]. Likewise, it is well recognized that initiating exercise training (where bone experiences increases in strain above normal levels of activity) is followed by increases in bone formation [82]. As just one example, classic exercise physiology studies showcase major differences in bone size and density in dominant versus non-dominant arms in lifetime tennis players [83,84]. Additional exercise-related factors (such as the number of loading cycles, the type of force applied, and changes in force over time) also influence skeletal adaptations [82,85,86,87] and collectively inform specific exercise recommendations to optimize skeletal health (see Table 1).

That said, exercise can have mechanical and metabolic effects on bone, with emerging data suggesting exercise may actually elicit an acute catabolic bone response [88,89,90,91,92,93,94,95,96]. In light of the underwhelming RCT signal for exercise to mitigate CR-induced bone loss, this section explores exercise-induced acute bone resorption as a plausible, yet underappreciated, mechanism to explain bone changes with exercise. It will begin with an overview of the effects of an acute bout of exercise followed by the response to chronic exercise training. This section is not intended, however, to serve as an exhaustive review of the mechanisms that may influence bone is response to CR and/or WL. For additional information on potential mechanisms, we refer the interested reader to several review articles [23,26,76].

### 3.1. Acute Exercise Effects on Markers of Bone Resorption and Bone Formation

The data on the bone biomarker response to exercise have demonstrated that a single, acute exercise bout can markedly increase bone resorption, as estimated using blood biomarkers. Changes in bone biomarkers in response to an acute exercise bout have been captured in young and older men and women during both weight-supported (e.g., cycling) [90,91,92,93,94,96] and weight-bearing (e.g., walking) endurance exercise [88,89,95,97,98,99,100]. These studies have captured robust changes in c-telopeptide of type I collagen (CTX; a marker of bone resorption) that appear to accompany a decrease in serum ionized calcium (iCa) and an increase in parathyroid hormone (PTH) [88,90,91,92,94,95,96,97,99,100]. The subsequent increase in PTH in response to the decrease in iCa appears to be an appropriate counter-regulatory mechanism to defend iCa during exercise. Because bone is the largest source of calcium storage in the body [101], bone resorption (as estimated by CTX) would be an effective means to quickly release calcium into the bloodstream [102] to sustain muscle contraction [103] or other functions. Indeed, several studies have highlighted that the decrease in iCa during exercise is the impetus for the increase in CTX with exercise [90,92,94,96].

The exercise-induced increase in CTX persists across the lifespan, although the magnitude of change is different between young [88,90,91,92,93,96,99,100,104] and older adults [93,95,97]. This may be a function of exercise intensity or exercise mode (weight-bearing versus weight-supported), which both have important implications for exercise prescription [82]. For example, in studies of young versus older adults at the same relative intensity (~75–80% of measured heart rate maximum) and the same duration (one hour), increases in CTX were +0.11 to +0.25 ng/mL [90,93,94] and +0.08 to +0.15 ng/mL [95,97] for young and older adults, respectively. However, it is important to note that absolute exercise intensity differed between studies of young and older adults. These studies also used a different mode of exercise, which could explain differences in magnitude of change for the measured biomarkers.

Bone remodeling is a coupled process, whereas bone formation follows the activation of bone resorption [105]. Thus, it would be assumed that the increase in CTX in response to acute exercise should also lead to an increase in bone formation. However, data have not shown a comparable increase in procollagen of type 1 n-terminal propeptide (P1NP; a marker of bone formation) in response to an acute exercise bout [92,96], but these studies have only collected measures to a maximum of four hours after exercise. This relatively short sampling timeline limits the ability to detect changes in P1NP in response to an acute exercise bout, as bone formation can be activated hours to days following a stimulus [106]. There is some evidence of an increase in P1NP from before to after exercise in response to 60-min of treadmill running in young men [89,99], but there is a lack of consistency in the bone formation response to exercise like what exists for the bone resorption response. The lack of agreement across studies could be due to sampling timelines, lab-to-lab differences, or mode of exercise. The previous studies that have found limited effect on P1NP used cycling exercise [92,96], whereas those reporting significant changes in P1NP utilized treadmill running [89,99]. Of importance, however, is that the available data on the bone formation response to an acute exercise about are from young adults; it is not known if there are any age-related changes that could disadvantage older adults.

The changes in CTX and P1NP have been primarily characterized in response to an acute endurance exercise bout [88,90,91,92,93,94,95,96,97,99,100,107], but emerging evidence suggests that there may be a similar CTX response to jumping exercises [108,109]. The nature of strength and power exercise (i.e., short bursts of activity with rest intervals) is closer to the type of exercise recommended to improve bone health [82,110,111,112,113,114], which may result in greater bone formation due to mechanical loading or perhaps less bone resorption if rest intervals reduce the decrease in iCa observed with endurance exercise.

### 3.2. Chronic Exercise Training Effects on Markers of Bone Resorption and Bone Formation

Results from chronic exercise training largely show that there is little to no effect on markers of bone resorption [115,116,117,118]. This is not entirely surprising, as there is not a clear mechanism whereby exercise training should reduce the overall amount of bone resorption in normally active, healthy individuals. It would be expected that any increases in aBMD, bone strength, or bone structure would instead come from increased bone formation versus a reduction in bone resorption.

If bone does respond to mechanical loading by increasing bone formation, exercise training should result in resting concentrations of markers of bone formation increasing over time. Indeed, it does appear that exercise training results in an increase in markers of bone formation [115,116,117,118,119,120,121,122]. However, there is a large degree of variability in the duration of exercise training (weeks versus months), type of exercise performed (endurance, resistance, meditative movement), and exercise intensity. The increases in P1NP observed in response to exercise training (~25% in response to 4–8 weeks of exercise training) [115,116] are also small compared to the observed increases in P1NP with anabolic osteoporosis medications (>90% at 4 weeks) [123,124] and may not be sufficient to counteract the increase in bone resorption in response to acute exercise or CR.

From the data available, it does not appear that mode of exercise (i.e., weight-bearing versus weight-supported) is an important determining factor in the bone resorption or formation response, unlike what has been observed for acute exercise bouts. However, there are many differences between studies that make the identification of the “best” type of exercise with respect to the effect on bone biomarkers challenging. Variations in the frequency and duration of exercise between studies may be potentially masking any differences in markers of bone formation by exercise mode in comparison with chronic exercise training. Further, bone biomarker assessment alone cannot quantify changes in bone mass, strength, or structure over the course of an intervention. While observed increases in a marker of bone formation in response to an intervention may provide some mechanistic insight into changes in bone modeling or remodeling, other outcomes, such as imaging, should be incorporated for a more complete picture of the bone response to exercise. This is especially relevant when the exercise performed has high degrees of mechanical loading, as observed with resistance training.

### 3.3. Summary

Acute exercise alone appears to result in a robust activation of bone resorption (as estimated by CTX), without a similar activation of bone formation. In contrast, long-term exercise training does appear to increase bone formation (as estimated by P1NP) over the course of an intervention; however, these increases tend to be small. Taken together, the acute activation of bone resorption combined with the modest increases in bone formation with training may explain why exercise is not more effective than observed in reducing CR-induced bone loss. As summarized previously, limited RCT data suggest that RT may be the preferred modality to preserve bone mass during CR; however, data on the bone biomarker response by exercise type (i.e., aerobic vs. resistance) are limited and warrant further investigation. A better understanding of the effect of CR and exercise on bone remodeling should help optimize WL strategies aimed at maximizing cardiometabolic benefit, while minimizing musculoskeletal harm, in older adults.

## 4. Conclusions and Future Research Directions

In summary, our structured literature review of RCTs specifically designed to assess the ability of exercise to mitigate CR-induced bone loss in older adults suggests a minimal osteoprotective effect (at best), with the strongest evidence in support of RT to modestly offset bone loss at the hip region. As presented in the narrative component of this review, one potential mechanism for the diminished effect of exercise in preserving bone mass during CR is exercise-induced bone resorption. Specifically, acute exercise bouts appear to increase bone resorption with minimal impact on bone formation. In contrast, chronic exercise training studies indicate that bone formation may increase over time; however, more data are needed before definite conclusions can be drawn.

Knowledge in this area would also be advanced by measurement of compartmental bone changes in RCTs of CR and exercise. Indeed, where bone is deposited (or lost) is a contributing factor to bone strength and structure and may also represent differential biological mechanisms between CR and exercise. In studies of weightlessness, for instance, endosteal resorption of bone predominates (although there is also some evidence on periosteal expansion, perhaps as a counter-regulatory mechanism to preserve bone strength) [125]. Alternatively, and though observed mainly in animal models, the strains experienced during exercise seem to mainly result in periosteal expansion [126]. Certainly, more research in this area is needed, particularly in older, clinical populations, as are future studies aimed at refining intervention delivery (including optimizing the frequency, intensity, timing, and type of exercise prescription, as well as the magnitude of prescribed CR deficit). Finally, consideration of alternative or adjuvant osteoprotective strategies (including anti-resorptive or anabolic osteoporosis medications) to mitigate CR-induced bone loss certainly seems warranted, particularly among older adults with obesity who would benefit from WL.

## Figures and Tables

**Table 1:** Exercise recommendations for older adults to promote bone health.

Modality	Frequency	Intensity	Example Exercises
Resistance Training *	≥2 days/week	≥2 set of 8–12 repetitions 1–3 min rest intervals 1 exercise per major muscle group * Can progress to high-velocity contractions with low to moderate intensity (30–70% maximum).	Compound exercises including (but not limited to): squats, lunges, hip ab/adduction, leg press, thoracic/lumbar extension, abdominal/postural exercises, or bent-over row.
Impact Training *	4–7 days/week	3–5 sets of 10–20 repetitions 2–4x body weight 1–2 min rest intervals 50–100 jumps per session. * Begin with RT and progress to impact training after 6–12 weeks.	Jump, hop, skipping, and stepping derivatives. Intensity can be increased via increased jump/step height or via external resistance (e.g., weighted vest)
Balance/Mobility Training *	≥2–3 h/week	Start with static exercises and progress to dynamic exercises. 15–20 min/day	Static: One-legged stand, tandem stand, etc. Dynamic: walking on toes, backwards walking, etc.
Aerobic Training	3–5 days/week	Moderate to vigorous 30–60 min/day	Walking, dancing, etc.

Recommendations derived from select working group publications [50,51,52]; * indicates emphasized exercise modality. RT—resistance training.

**Table 2:** Design characteristics of randomized controlled trials assessing the ability of exercise to mitigate caloric restriction-induced reductions in dual-energy x-ray absorptiometry- (DXA) acquired areal bone mineral density (aBMD) among older adults.

Author (Year)	N (% Female)	Age (Years)	BMI (kg/m^2^)	Duration (Months)	Intervention Characteristics			WL (%)	DXA aBMD Percent Change from Baseline			
					Group	*n*	Brief Description of Intervention		TB	LS	TH	FN
Svendsen et al. (1993) [61]	121 (100)	54	29.7	3	Control	21	Maintained normal lifestyle	0.7^β^	−1.2	−0.4	--	--
					CR	51	~1000 kcal/day target	−12.2	−1.9	−1.6	--	--
					CR + EX	49	~1000 kcal/day target + RT and AT 3 d/week	−13.2	−1.9	−2.4^↓^	--	--
Daly et al. (2005) [62]	29 (45)	67	32	0 to 6 6 to 12	CR	13	Mo. 0–6: 0.25 kg WL/wk Mo. 6–12: no dietary guidance	−3.3^↓^ 1.6^↑^	−0.9^↓β^ −0.6^↓^	−0.6 −1.5	-- --	−1.1 −1.0
					CR + RT	16	Mo. 0–6: 0.25 kg WL/week + center-based RT 3 d/week Mo. 6–12: No dietary guidance + at-home RT 3 d/week	−2.7^↓^ 1.7^↑^	−0.3 −0.4	−0.1 −0.7	-- --	−0.5 −0.4
Villareal et al. (2011) [63]	107 (63)	70	37	12	Control	27	Maintained normal lifestyle	<−1.0 ^β^	0.5	0.6	−0.7 ^↓D^	0.0
Shah et al. (2011) [64]					CR	26	~500–750 kcal/day deficit	−10.0^↓^	−0.4	0.9	−2.6^↓^	−2.1^↓C^
					EX	26	Multimodal exercise (AT, RT, FT, BT) 3 d/wk	−1.0 ^β^	0.6	1.0	1.4 ^↑β^	1.2^D^
					CR + EX	28	~500–750 kcal/day deficit + multimodal exercise 3 d/wk	−9.0^↓^	0.8	0.7	−1.1^↓^	−0.9^E^
Beavers et al. (2014) [65]	284 (74)	66	33	18	EX	95	Multimodal exercise (AT, RT) 3 d/week.	−1.4^↓^	--	0.7	−0.2^β^	−0.1^β^
					CR	88	~800–1000 kcal/day deficit	−9.7^↓^	--	0.5	−2.4	−1.7
					CR + EX	101	~800–1000 kcal/day deficit + Walk + RE + Walk 3 d/week	−10.4^↓^	--	0.1	−2.0	−1.6
Weiss et al. (2017) [66]	52 (75)	57	27.7	3.5	CR	17	20% energy deficit for 12–14 wks	−6.9^↓^	0.1	0.3	−0.3	--
					EX	16	60 min AT ~7 d/week	−7.2^↓^	−0.1	1.1	−0.7	--
					CR + EX	19	Half of CR and EX prescriptions	−7.4^↓^	−0.4	−0.9	0.0	--
Beavers et al. (2018) [67]	187 (70)	67	34.5	18	CR	60	~330 kcal/day deficit	−5.9^↓β^	--	1.0	−2.2	−0.9
					CR + AT	67	~330 kcal/day deficit + 45 min AT 4 d/week	−10.6^↓^	--	−0.5	−2.7	0.4
					CR + RT	60	~330 kcal + RT 4 day/week	−10.4^↓^	--	0.6	−2.5	0.4

Notes. BMI—body mass index; WL—weight loss; TB—total body; LS—lumbar spine; TH—total hip; FN—femoral neck; EX—multimodal exercise (e.g., AT + RT) AT—aerobic training; RT—resistance training; FT—flexibility training; BT—balance training. ↑ or ↓: significant increase or decrease from baseline (all *p* ≤ 0.05); β: significant between group difference (all *p* ≤ 0.05). Villareal/Shah 2011: C—different from control group; D—different from diet group; E—different from exercise group.

## Abbreviations

aBMD	Areal bone mineral density
AT	Aerobic training
BMI	Body mass index
CR	Caloric restriction
CTX	C-telopeptide of type I collagen
DXA	Dual-energy x-ray absorptiometry
EX	Exercise
GRECC	Geriatric Research, Education, and Clinical Center
P1NP	Procollagen of type 1 n-terminal propeptide
PRISMA	Preferred Reporting Items for Systematic reviews and Meta-Analyses
PTH	Parathyroid hormone
QCT	Quantitative computed tomography
RCT	Randomized controlled trial
RT	Resistance training
WL	Weight loss

## Appendix A. Search Strategy Used to Identify RCTs Included in the Structured Component of the Review

**PubMed search strategy**

(((((“Exercise”[Mesh]) OR (“Physical Exertion”[Mesh])) OR (“Physical Fitness”[Mesh])) OR (exercis*[Title/Abstract] OR “physical activit*”[Title/Abstract] OR exert*[Title/Abstract] OR “physical fitness”[Title/Abstract])) AND ((“Weight Loss”[Mesh]) OR (weight loss[Title/Abstract] OR weight losing[Title/Abstract] OR weight reduc*[Title/Abstract] OR weight decreas*[Title/Abstract]))) AND (((bone and bones[MeSH Terms]) OR (bone density[MeSH Terms])) OR (bone*[Title/Abstract] OR “bone densit*”[Title/Abstract] OR “bone mineral densit*”[Title/Abstract] OR “bone mineral content”[Title/Abstract] OR “bone mineral”[Title/Abstract] OR “bone loss”[Title/Abstract] OR “bone mass”[Title/Abstract] OR “bone strength”[Title/Abstract] OR BMC[Title/Abstract] OR BMD[Title/Abstract])) NOT (mouse[Title/Abstract] OR mice[Title/Abstract] OR rat[Title/Abstract] OR rats[Title/Abstract] OR rabbit[Title/Abstract] OR rabbits[Title/Abstract] OR Leporidae[Title/Abstract] OR horse[Title/Abstract] OR horses[Title/Abstract] OR Equidae[Title/Abstract]) Filters: Randomized Controlled Trial, Middle Aged + Aged: 45+ years

49 results.

**Science Citation Index in Web of Science search strategy**

(TS = (exercis* OR physical activit* OR exert*) AND TS = (“weight loss” OR weight losing OR weight reduc* OR weight decreas*) AND TS= (bone densit* OR “bone mineral densit*” OR “bone mineral content” OR “bone loss” OR “bone mass” OR “bone mineral” OR “bone strength” OR BMC OR BMD) AND TS = (“randomi* controlled trial” OR “single blind*” OR “double blind*”)) NOT AB = (mouse OR mice OR rat OR rats OR rabbit OR rabbits OR Leporidae OR horse OR horses OR Equidae)

Refined by: DOCUMENT TYPES: (ARTICLE) Timespan: 1945–2020.

Indexes: SCI-EXPANDED.

166 results.

**Table A1:** Embase search strategy.

1	exercise’/exp
2	physical activity’/exp
3	(exercise:ti,ab,kw OR ‘physical activity, capacity’:ti,ab,kw) AND performance:ti,ab,kw
4	#1 OR #2 OR #3
5	body weight loss’/exp
6	weight loss’:ti,ab,kw OR ‘weight losing’:ti,ab,kw OR ‘weight reduc*’:ti,ab,kw OR ‘weight decreas*’:ti,ab,kw
7	#5 OR #6
8	bone’/exp
9	bone density’/exp
10	bone*:ti,ab,kw OR ‘bone densit*’:ti,ab,kw OR ‘bone mineral’:ti,ab,kw OR ‘bone loss’:ti,ab,kw OR ‘bone mass’:ti,ab,kw OR bmc:ti,ab,kw OR bmd:ti,ab,kw
11	#8 OR #9 OR #10
12	#4 AND #7 AND #11
13	#12 AND (‘randomized controlled trial’/de) AND ([aged]/lim OR [middle aged]/lim OR [very elderly]/lim)
14	(mouse:ti,ab,kw OR rat:ti,ab,kw OR leporidae:ti,ab,kw OR equidae:ti,ab,kw)
15	#13 NOT #14

50 results.

**Table A2:** SportDiscus in EbscoHost search strategy.

1	TI (exercis* or physical activit* or exert* or physical fitness) OR AB (exercis* or physical activit* or exert* or physical fitness) OR KW (exercis* or physical activit* or exert* or physical fitness)
2	TI (weight loss or losing weight or weight reduc* or weight decreas* or lose weight or weight management) OR AB (weight loss or losing weight or weight reduc* or weight decreas* or lose weight or weight management) OR KW (weight loss or losing weight or weight reduc* or weight decreas* or lose weight or weight management)
3	TI (bone* or bone density or bone mineral density or bone mineral content OR bone loss OR bone mass OR bone strength OR BMC OR BMD) OR AB (bone* or bone density or bone mineral density or bone mineral content OR bone loss OR bone mass OR bone strength OR BMC OR BMD) OR KW (bone* or bone density or bone mineral density or bone mineral content or bone loss or bone mass or bone strength OR BMC OR BMD)
4	TI (“randomi* controlled trial” OR “single blind*” OR “double blind*”) OR AB (“randomi* controlled trial” OR “single blind*” OR “double blind*”) OR KW (“randomi* controlled trial” OR “single blind*” OR “double blind*”)
5	#1 AND #2 AND #3 AND #4

2 results.

## References

[1] Anderson L.A., Goodman R.A., Holtzman D., Posner S.F., Northridge M.E. (2012). Aging in the United States: Opportunities and challenges for public health. Am. J. Public Health.

[2] Wang Y.C., McPherson K., Marsh T., Gortmaker S.L., Brown M. (2011). Health and economic burden of the projected obesity trends in the USA and the UK. Lancet.

[3] Nguyen T.V., Sambrook P.N., Eisman J.A. (1998). Bone loss, physical activity, and weight change in elderly women: The Dubbo Osteoporosis Epidemiology Study. J. Bone Miner Res..

[4] Lang T.F., Sigurdsson S., Karlsdottir G., Oskarsdottir D., Sigmarsdottir A., Chengshi J., Kornak J., Harris T.B., Sigurdsson G., Jonsson B.Y. (2012). Age-related loss of proximal femoral strength in elderly men and women: The Age Gene/Environment Susceptibility Study—Reykjavik. Bone.

[5] Zhu K., Hunter M., James A., Lim E.M., Walsh J.P. (2015). Associations between body mass index, lean and fat body mass and bone mineral density in middle-aged Australians: The Busselton Healthy Ageing Study. Bone.

[6] Compston J.E., Watts N.B., Chapurlat R., Cooper C., Boonen S., Greenspan S., Pfeilschifter J., Silverman S., Diez-Perez A., Lindsay R. (2011). Obesity is not protective against fracture in postmenopausal women: GLOW. Am. J. Med..

[7] Compston J. (2013). Obesity and Bone. Curr. Osteoporos. Rep..

[8] Compston J. (2015). Obesity and fractures in postmenopausal women. Curr. Opin. Rheumatol..

[9] Nielson C.M., Srikanth P., Orwoll E.S. (2012). Obesity and fracture in men and women: An epidemiologic perspective. J. Bone Miner Res..

[10] Sukumar D., Schlussel Y., Riedt C.S., Gordon C., Stahl T., Shapses S.A. (2011). Obesity alters cortical and trabecular bone density and geometry in women. Osteoporos. Int..

[11] Nielson C.M., Marshall L.M., Adams A.L., LeBlanc E.S., Cawthon P.M., Ensrud K., Stefanick M.L., Barrett-Connor E., Orwoll E.S. (2011). Osteoporotic Fractures in Men Study Research, BMI and fracture risk in older men: The osteoporotic fractures in men study (MrOS). J. Bone Miner Res..

[12] Premaor M.O., Pilbrow L., Tonkin C., Parker R.A., Compston J. (2010). Obesity and fractures in postmenopausal women. J. Bone Miner Res..

[13] Cockerill W., Lunt M., Silman A.J., Cooper C., Lips P., Bhalla A.K., Cannata J.B., Eastell R., Felsenberg D., Gennari C. (2004). Health-related quality of life and radiographic vertebral fracture. Osteoporos. Int..

[14] Center J.R., Nguyen T.V., Schneider D., Sambrook P.N., Eisman J.A. (1999). Mortality after all major types of osteoporotic fracture in men and women: An observational study. Lancet.

[15] Office of the Surgeon General (US) (2004). The Burden of Bone Disease. Bone Health and Osteoporosis: A Report of the Surgeon General.

[16] Colon-Emeric C.S., Saag K.G. (2006). Osteoporotic fractures in older adults. Best Pract. Res. Clin. Rheumatol..

[17] Peck W.A. (1993). Consensus development conference: Diagnosis, prophylaxis, and treatment of osteoporosis. Am. J. Med..

[18] Ray N.F., Chan J.K., Thamer M., Melton L.J. (1997). Medical expenditures for the treatment of osteoporotic fractures in the United States in 1995: Report from the National Osteoporosis Foundation. J. Bone Miner Res..

[19] Ensrud K.E., Fullman R.L., Barrett-Connor E., Cauley J.A., Stefanick M.L., Fink H.A., Lewis C.E., Orwoll E. (2005). Voluntary weight reduction in older men increases hip bone loss: The osteoporotic fractures in men study. J. Clin. Endocrinol. Metab..

[20] Dennison E., Eastell R., Fall C.H., Kellingray S., Wood P.J., Cooper C. (1999). Determinants of bone loss in elderly men and women: A prospective population-based study. Osteoporos. Int..

[21] Hannan M.T., Felson D.T., Anderson J.J. (1992). Bone mineral density in elderly men and women: Results from the Framingham osteoporosis study. J. Bone Miner Res..

[22] Knoke J.D., Barrett-Connor E. (2003). Weight loss: A determinant of hip bone loss in older men and women. The Rancho Bernardo Study. Am. J. Epidemiol..

[23] Shapses S.A., Sukumar D. (2012). Bone metabolism in obesity and weight loss. Annu. Rev. Nutr..

[24] Zibellini J., Seimon R.V., Lee C.M., Gibson A.A., Hsu M.S., Shapses S.A., Nguyen T.V., Sainsbury A. (2015). Does Diet-Induced Weight Loss Lead to Bone Loss in Overweight or Obese Adults? A Systematic Review and Meta-Analysis of Clinical Trials. J. Bone Miner Res..

[25] Villareal D.T., Fontana L., Das S.K., Redman L., Smith S.R., Saltzman E., Bales C., Rochon J., Pieper C., Huang M. (2016). Effect of Two-Year Caloric Restriction on Bone Metabolism and Bone Mineral Density in Non-Obese Younger Adults: A Randomized Clinical Trial. J. Bone Miner Res..

[26] Iwaniec U.T., Turner R.T. (2016). Influence of body weight on bone mass, architecture and turnover. J. Endocrinol..

[27] Schwartz A.V., Johnson K.C., Kahn S.E., Shepherd J.A., Nevitt M.C., Peters A.L., Walkup M.P., Hodges A., Williams C.C., Bray G.A. (2012). Effect of 1 year of an intentional weight loss intervention on bone mineral density in type 2 diabetes: Results from the Look AHEAD randomized trial. J. Bone Miner Res..

[28] Lipkin E.W., Schwartz A.V., Anderson A.M., Davis C., Johnson K.C., Gregg E.W., Bray G.A., Berkowitz R., Peters A.L., Hodges A. (2014). The Look AHEAD Trial: Bone loss at 4-year follow-up in type 2 diabetes. Diabetes Care.

[29] Ensrud K.E., Ewing S.K., Stone K.L., Cauley J.A., Bowman P.J., Cummings S.R. (2003). Study of Osteoporotic Fractures Research, G. Intentional and unintentional weight loss increase bone loss and hip fracture risk in older women. J. Am. Geriatr. Soc..

[30] Crandall C.J., Yildiz V.O., Wactawski-Wende J., Johnson K.C., Chen Z., Going S.B., Wright N.C., Cauley J.A. (2015). Postmenopausal weight change and incidence of fracture: Post hoc findings from Women’s Health Initiative Observational Study and Clinical Trials. BMJ.

[31] Johnson K.C., Bray G.A., Cheskin L.J., Clark J.M., Egan C.M., Foreyt J.P., Garcia K.R., Glasser S., Greenway F.L., Gregg E.W. (2017). The Effect of Intentional Weight Loss on Fracture Risk in Persons With Diabetes: Results From the Look AHEAD Randomized Clinical Trial. J. Bone Miner Res..

[32] Lv Q.-B., Fu X., Jin H.-M., Xu H.-C., Huang Z.-Y., Xu H.-Z., Chi Y.-L., Wu A.-M. (2015). The relationship between weight change and risk of hip fracture: Meta-analysis of prospective studies. Sci. Rep..

[33] Langlois J.A., Visser M., Davidovic L.S., Maggi S., Li G., Harris T.B. (1998). Hip fracture risk in older white men is associated with change in body weight from age 50 years to old age. Arch. Intern. Med..

[34] Ensrud K.E., Cauley J., Lipschutz R., Cummings S.R. (1997). Weight change and fractures in older women. Study of Osteoporotic Fractures Research Group. Arch. Intern. Med..

[35] Soltani S., Hunter G.R., Kazemi A., Shab-Bidar S. (2016). The effects of weight loss approaches on bone mineral density in adults: A systematic review and meta-analysis of randomized controlled trials. Osteoporos. Int..

[36] Villalon K.L., Gozansky W.S., Van Pelt R.E., Wolfe P., Jankowski C.M., Schwartz R.S., Kohrt W.M. (2011). A losing battle: Weight regain does not restore weight loss-induced bone loss in postmenopausal women. Obesity.

[37] Avenell A., Richmond P.R., Lean M.E., Reid D.M. (1994). Bone loss associated with a high fibre weight reduction diet in postmenopausal women. Eur. J. Clin. Nutr..

[38] Park S.W., Goodpaster B.H., Strotmeyer E.S., Kuller L.H., Broudeau R., Kammerer C., de Rekeneire N., Harris T.B., Schwartz A.V., Tylavsky F.A. (2007). Accelerated loss of skeletal muscle strength in older adults with type 2 diabetes: The health, aging, and body composition study. Diabetes Care.

[39] Waters D.L., Vawter R., Qualls C., Chode S., Armamento-Villareal R., Villareal D.T. (2013). Long-term maintenance of weight loss after lifestyle intervention in frail, obese older adults. J. Nutr. Health Aging.

[40] Kammire D.E., Walkup M.P., Ambrosius W.T., Lenchik L., Shapses S.A., Nicklas B.J., Houston D.K., Marsh A.P., Rejeski W.J., Beavers K.M. (2019). Effect of Weight Change Following Intentional Weight Loss on Bone Health in Older Adults with Obesity. Obesity.

[41] Von Thun N.L., Sukumar D., Heymsfield S.B., Shapses S.A. (2014). Does bone loss begin after weight loss ends? Results 2 years after weight loss or regain in postmenopausal women. Menopause.

[42] Hinton P.S., Rector R.S., Linden M.A., Warner S.O., Dellsperger K.C., Chockalingam A., Whaley-Connell A.T., Liu Y., Thomas T.R. (2012). Weight-loss-associated changes in bone mineral density and bone turnover after partial weight regain with or without aerobic exercise in obese women. Eur. J. Clin. Nutr..

[43] Villareal D.T., Apovian C.M., Kushner R.F., Klein S., American Society for Nutrition and NAASO (2005). Obesity in older adults: Technical review and position statement of the American Society for Nutrition and NAASO, The Obesity Society. Obes. Res..

[44] Shapses S.A., Sukumar D., Schneider S.H., Schlussel Y., Sherrell R.M., Field M.P., Ambia-Sobhan H. (2013). Vitamin D supplementation and calcium absorption during caloric restriction: A randomized double-blind trial. Am. J. Clin. Nutr..

[45] Ricci T.A., Chowdhury H.A., Heymsfield S.B., Stahl T., Pierson R.N., Shapses S.A. (1998). Calcium supplementation suppresses bone turnover during weight reduction in postmenopausal women. J. Bone Miner Res..

[46] Sukumar D., Ambia-Sobhan H., Zurfluh R., Schlussel Y., Stahl T.J., Gordon C.L., Shapses S.A. (2011). Areal and volumetric bone mineral density and geometry at two levels of protein intake during caloric restriction: A randomized, controlled trial. J. Bone Miner Res..

[47] Bolam K.A., Skinner T.L., Jenkins D.G., Galvao D.A., Taaffe D.R. (2015). The Osteogenic Effect of Impact-Loading and Resistance Exercise on Bone Mineral Density in Middle-Aged and Older Men: A Pilot Study. Gerontology.

[48] Martyn-St James M., Carroll S. (2010). Effects of different impact exercise modalities on bone mineral density in premenopausal women: A meta-analysis. J. Bone Miner. Metab..

[49] Zhao R., Zhao M., Xu Z. (2015). The effects of differing resistance training modes on the preservation of bone mineral density in postmenopausal women: A meta-analysis. Osteoporos. Int..

[50] Giangregorio L.M., Papaioannou A., Macintyre N.J., Ashe M.C., Heinonen A., Shipp K., Wark J., McGill S., Keller H., Jain R. (2014). Too Fit To Fracture: Exercise recommendations for individuals with osteoporosis or osteoporotic vertebral fracture. Osteoporos. Int..

[51] Beck B.R., Daly R.M., Singh M.A., Taaffe D.R. (2017). Exercise and Sports Science Australia (ESSA) position statement on exercise prescription for the prevention and management of osteoporosis. J. Sci. Med. Sport.

[52] Daly R.M., Dalla Via J., Duckham R.L., Fraser S.F., Helge E.W. (2019). Exercise for the prevention of osteoporosis in postmenopausal women: An evidence-based guide to the optimal prescription. Braz. J. Phys. Ther..

[53] Sherrington C., Fairhall N.J., Wallbank G.K., Tiedemann A., Michaleff Z.A., Howard K., Clemson L., Hopewell S., Lamb S.E. (2019). Exercise for preventing falls in older people living in the community. Cochrane Database Syst. Rev..

[54] Piercy K.L., Troiano R.P., Ballard R.M., Carlson S.A., Fulton J.E., Galuska D.A., George S.M., Olson R.D. (2018). The Physical Activity Guidelines for Americans. JAMA.

[55] Hosny I.A., Elghawabi H.S., Younan W.B., Sabbour A.A., Gobrial M.A. (2012). Beneficial impact of aerobic exercises on bone mineral density in obese premenopausal women under caloric restriction. Skeletal Radiol..

[56] Nakata Y., Ohkawara K., Lee D.J., Okura T., Tanaka K. (2008). Effects of additional resistance training during diet-induced weight loss on bone mineral density in overweight premenopausal women. J. Bone Miner. Metab..

[57] Ryan A.S., Nicklas B.J., Dennis K.E. (1998). Aerobic exercise maintains regional bone mineral density during weight loss in postmenopausal women. J. Appl. Physiol..

[58] Villareal D.T., Fontana L., Weiss E.P., Racette S.B., Steger-May K., Schechtman K.B., Klein S., Holloszy J.O. (2006). Bone mineral density response to caloric restriction-induced weight loss or exercise-induced weight loss: A randomized controlled trial. Arch. Intern. Med..

[59] Villareal D.T., Shah K., Banks M.R., Sinacore D.R., Klein S. (2008). Effect of Weight Loss and Exercise Therapy on Bone Metabolism and Mass in Obese Older Adults: A One-Year Randomized Controlled Trial. J. Clin. Endocrinol. Metab..

[60] Anderson R.E., Wadden T.A., Herzog R.J. (1997). Changes in bone mineral content in obese dieting women. Metabolism.

[61] Svendsen O.L., Hassager C., Christiansen C. (1993). Effect of an energy-restrictive diet, with or without exercise, on lean tissue mass, resting metabolic rate, cardiovascular risk factors, and bone in overweight postmenopausal women. Am. J. Med..

[62] Daly R.M., Dunstan D.W., Owen N., Jolley D., Shaw J.E., Zimmet P.Z. (2005). Does high-intensity resistance training maintain bone mass during moderate weight loss in older overweight adults with type 2 diabetes?. Osteoporos. Int..

[63] Villareal D.T., Chode S., Parimi N., Sinacore D.R., Hilton T., Armamento-Villareal R., Napoli N., Qualls C., Shah K. (2011). Weight loss, exercise, or both and physical function in obese older adults. N. Engl. J. Med..

[64] Shah K., Armamento-Villareal R., Parimi N., Chode S., Sinacore D.R., Hilton T.N., Napoli N., Qualls C., Villareal D.T. (2011). Exercise training in obese older adults prevents increase in bone turnover and attenuates decrease in hip bone mineral density induced by weight loss despite decline in bone-active hormones. J. Bone Miner Res..

[65] Beavers D.P., Beavers K.M., Loeser R.F., Walton N.R., Lyles M.F., Nicklas B.J., Shapses S.A., Newman J.J., Messier S.P. (2014). The independent and combined effects of intensive weight loss and exercise training on bone mineral density in overweight and obese older adults with osteoarthritis. Osteoarthr. Res. Soc..

[66] Weiss E.P., Jordan R.C., Frese E.M., Albert S.G., Villareal D.T. (2017). Effects of Weight Loss on Lean Mass, Strength, Bone, and Aerobic Capacity. Med. Sci. Sports Exerc..

[67] Beavers K.M., Walkup M.P., Weaver A.A., Lenchik L., Kritchevsky S.B., Nicklas B.J., Ambrosius W.T., Stitzel J.D., Register T.C., Shapses S.A. (2018). Effect of Exercise Modality during Weight Loss on Bone Health in Older Adults With Obesity and Cardiovascular Disease or Metabolic Syndrome: A Randomized Controlled Trial. J. Bone Miner Res..

[68] Bouxsein M.L., Eastell R., Lui L.Y., Wu L.A., de Papp A.E., Grauer A., Marin F., Cauley J.A., Bauer D.C., Black D.M. (2019). Change in Bone Density and Reduction in Fracture Risk: A Meta-Regression of Published Trials. J. Bone Miner Res..

[69] Black D.M., Bauer D.C., Vittinghoff E., Lui L.Y., Grauer A., Marin F., Khosla S., de Papp A., Mitlak B., Cauley J.A. (2020). Treatment-related changes in bone mineral density as a surrogate biomarker for fracture risk reduction: Meta-regression analyses of individual patient data from multiple randomised controlled trials. Lancet Diabetes Endocrinol..

[70] Heaney R.P. (2009). The bone-remodeling transient: Implications for the interpretation of clinical studies of bone mass change. J. Bone Miner Res..

[71] Messier S.P., Legault C., Mihalko S., Miller G.D., Loeser R.F., DeVita P., Lyles M., Eckstein F., Hunter D.J., Williamson J.D. (2009). The intensive diet and exercise for arthritis (IDEA) trial: Design and rationale. BMC Musculoskelet. Disord..

[72] Villareal D.T., Aguirre L., Gurney A.B., Waters D.L., Sinacore D.R., Colombo E., Armamento-Villareal R., Qualls C. (2017). Aerobic or Resistance Exercise, or Both, in Dieting Obese Older Adults. N. Engl. J. Med..

[73] Armamento-Villareal R., Aguirre L., Waters D.L., Napoli N., Qualls C., Villareal D.T. (2019). Effect of aerobic or resistance exercise, or both, on bone mineral density and bone metabolism in obese older adults while dieting: A randomized controlled trial. J. Bone Miner Res..

[74] Burton E., Hill A.M., Pettigrew S., Lewin G., Bainbridge L., Farrier K., Airey P., Hill K.D. (2017). Why do seniors leave resistance training programs?. Clin. Interv. Aging.

[75] Endo Y., Nourmahnad A., Sinha I. (2020). Optimizing Skeletal Muscle Anabolic Response to Resistance Training in Aging. Front Physiol..

[76] Turner C.H., Robling A.G. (2005). Mechanisms by which exercise improves bone strength. J. Bone Miner Metab..

[77] Christen P., Ito K., Ellouz R., Boutroy S., Sornay-Rendu E., Chapurlat R.D., van Rietbergen B. (2014). Bone remodelling in humans is load-driven but not lazy. Nat. Commun..

[78] Bass S.L., Eser P., Daly R. (2005). The effect of exercise and nutrition on the mechanostat. J. Musculoskelet. Neuronal Interact..

[79] Frost H.M. (1987). Bone “mass″ and the “mechanostat″: A proposal. Anat. Rec..

[80] Tagliaferri C., Wittrant Y., Davicco M.J., Walrand S., Coxam V. (2015). Muscle and bone, two interconnected tissues. Ageing Res. Rev..

[81] Fleischer J., Stein E.M., Bessler M., Della Badia M., Restuccia N., Olivero-Rivera L., McMahon D.J., Silverberg S.J. (2008). The decline in hip bone density after gastric bypass surgery is associated with extent of weight loss. J. Clin. Endocrinol. Metab..

[82] Turner C.H., Robling A.G. (2002). Designing exercise regimens to increase bone strength. Exerc. Sport Sci. Rev..

[83] Huddleston A.L., Rockwell D., Kulund D.N., Harrison R.B. (1980). Bone mass in lifetime tennis athletes. JAMA.

[84] Krahl H., Michaelis U., Pieper H.G., Quack G., Montag M. (1994). Stimulation of bone growth through sports. A radiologic investigation of the upper extremities in professional tennis players. Am. J. Sports Med..

[85] Robling A.G., Burr D.B., Turner C.H. (2001). Recovery periods restore mechanosensitivity to dynamically loaded bone. J. Exp. Biol..

[86] Robling A.G., Hinant F.M., Burr D.B., Turner C.H. (2002). Shorter, more frequent mechanical loading sessions enhance bone mass. Med. Sci. Sports Exerc..

[87] Robling A.G., Hinant F.M., Burr D.B., Turner C.H. (2002). Improved bone structure and strength after long-term mechanical loading is greatest if loading is separated into short bouts. J. Bone Miner Res..

[88] Scott J.P., Sale C., Greeves J.P., Casey A., Dutton J., Fraser W.D. (2010). The effect of training status on the metabolic response of bone to an acute bout of exhaustive treadmill running. J. Clin. Endocrinol. Metab..

[89] Scott J.P., Sale C., Greeves J.P., Casey A., Dutton J., Fraser W.D. (2012). Effect of fasting versus feeding on the bone metabolic response to running. Bone.

[90] Barry D.W., Hansen K.C., van Pelt R.E., Witten M., Wolfe P., Kohrt W.M. (2011). Acute calcium ingestion attenuates exercise-induced disruption of calcium homeostasis. Med. Sci. Sports Exerc..

[91] Barry D.W., Kohrt W.M. (2007). Acute effects of 2 hours of moderate-intensity cycling on serum parathyroid hormone and calcium. Calcif. Tissue Int..

[92] Kohrt W.M., Wherry S.J., Wolfe P., Sherk V.D., Wellington T., Swanson C.M., Weaver C.M., Boxer R.S. (2018). Maintenance of Serum Ionized Calcium During Exercise Attenuates Parathyroid Hormone and Bone Resorption Responses. J. Bone Miner Res..

[93] Kohrt W.M., Wolfe P., Sherk V.D., Wherry S.J., Wellington T., Melanson E.L., Swanson C.M., Weaver C.M., Boxer R.S. (2019). Dermal Calcium Loss Is Not the Primary Determinant of Parathyroid Hormone Secretion during Exercise. Med. Sci. Sports Exerc..

[94] Sherk V.D., Wherry S.J., Barry D.W., Shea K.L., Wolfe P., Kohrt W.M. (2017). Calcium Supplementation Attenuates Disruptions in Calcium Homeostasis during Exercise. Med. Sci. Sports Exerc..

[95] Wherry S.J., Swanson C.M., Wolfe P., Wellington T., Boxer R.S., Schwartz R.S., Kohrt W.M. (2019). Bone Biomarker Response to Walking under Different Thermal Conditions in Older Adults. Med. Sci. Sports Exerc..

[96] Haakonssen E.C., Ross M.L., Knight E.J., Cato L.E., Nana A., Wluka A.E., Cicuttini F.M., Wang B.H., Jenkins D.G., Burke L.M. (2015). The effects of a calcium-rich pre-exercise meal on biomarkers of calcium homeostasis in competitive female cyclists: A randomised crossover trial. PLoS ONE.

[97] Shea K.L., Barry D.W., Sherk V.D., Hansen K.C., Wolfe P., Kohrt W.M. (2014). Calcium supplementation and parathyroid hormone response to vigorous walking in postmenopausal women. Med. Sci. Sports Exerc..

[98] Chodzko-Zajko W.J., Proctor D.N., Fiatarone Singh M.A., Minson C.T., Nigg C.R., Salem G.J., Skinner J.S., American College of Sports Medicine (2009). American College of Sports Medicine position stand. Exercise and physical activity for older adults. Med. Sci. Sports Exerc..

[99] Scott J.P., Sale C., Greeves J.P., Casey A., Dutton J., Fraser W.D. (2011). The role of exercise intensity in the bone metabolic response to an acute bout of weight-bearing exercise. J. Appl. Physiol..

[100] Townsend R., Elliott-Sale K.J., Pinto A.J., Thomas C., Scott J.P., Currell K., Fraser W.D., Sale C. (2016). Parathyroid Hormone Secretion Is Controlled by Both Ionized Calcium and Phosphate During Exercise and Recovery in Men. J. Clin. Endocrinol. Metab..

[101] Grabowski P. (2009). Physiology of bone. Endocr. Dev..

[102] Silva B.C., Costa A.G., Cusano N.E., Kousteni S., Bilezikian J.P. (2011). Catabolic and anabolic actions of parathyroid hormone on the skeleton. J. Endocrinol. Investig..

[103] Szent-Gyorgyi A.G. (1975). Calcium regulation of muscle contraction. Biophys. J..

[104] Sherk V.D., Barry D.W., Villalon K.L., Hansen K.C., Wolfe P., Kohrt W.M. (2014). Bone loss over 1 year of training and competition in female cyclists. Clin. J. Sport Med..

[105] Hadjidakis D.J., Androulakis I.I. (2006). Bone remodeling. Ann. N. Y. Acad. Sci..

[106] Eriksen E.F. (2010). Cellular mechanisms of bone remodeling. Rev. Endocr. Metab. Disord..

[107] Scott J.P., Sale C., Greeves J.P., Casey A., Dutton J., Fraser W.D. (2014). Treadmill running reduces parathyroid hormone concentrations during recovery compared with a nonexercising control group. J. Clin. Endocrinol. Metab..

[108] Rantalainen T., Heinonen A., Linnamo V., Komi P.V., Takala T.E., Kainulainen H. (2009). Short-term bone biochemical response to a single bout of high-impact exercise. J. Sports Sci. Med..

[109] Prawiradilaga R.S., Madsen A.O., Jorgensen N.R., Helge E.W. (2020). Acute response of biochemical bone turnover markers and the associated ground reaction forces to high-impact exercise in postmenopausal women. Biol. Sport.

[110] Barry D.W., Kohrt W.M. (2008). Exercise and the preservation of bone health. J. Cardiopulm. Rehabil. Prev..

[111] Kelley G.A. (1998). Exercise and regional bone mineral density in postmenopausal women: A meta-analytic review of randomized trials. Am. J. Phys. Med. Rehabil..

[112] Kelley G.A., Kelley K.S., Kohrt W.M. (2013). Exercise and bone mineral density in men: A meta-analysis of randomized controlled trials. Bone.

[113] Kohrt W.M., Bloomfield S.A., Little K.D., Nelson M.E., Yingling V.R., American College of Sports M. (2004). American College of Sports Medicine Position Stand: Physical activity and bone health. Med. Sci. Sports Exerc..

[114] Alam I., Warden S.J., Robling A.G., Turner C.H. (2005). Mechanotransduction in bone does not require a functional cyclooxygenase-2 (COX-2) gene. J. Bone Miner Res..

[115] Adami S., Gatti D., Viapiana O., Fiore C.E., Nuti R., Luisetto G., Ponte M., Rossini M., Group B.S. (2008). Physical activity and bone turnover markers: A cross-sectional and a longitudinal study. Calcif. Tissue Int..

[116] Ardawi M.S., Rouzi A.A., Qari M.H. (2012). Physical activity in relation to serum sclerostin, insulin-like growth factor-1, and bone turnover markers in healthy premenopausal women: A cross-sectional and a longitudinal study. J. Clin. Endocrinol. Metab..

[117] Erickson C.R., Vukovich M.D. (2010). Osteogenic index and changes in bone markers during a jump training program: A pilot study. Med. Sci. Sports Exerc..

[118] Kim S., Bemben M.G., Knehans A.W., Bemben D.A. (2015). Effects of an 8-Month Ashtanga-Based Yoga Intervention on Bone Metabolism in Middle-Aged Premenopausal Women: A Randomized Controlled Study. J. Sports Sci. Med..

[119] Alghadir A.H., Aly F.A., Gabr S.A. (2014). Effect of Moderate Aerobic Training on Bone Metabolism Indices among Adult Humans. Pak. J. Med. Sci..

[120] Roghani T., Torkaman G., Movasseghe S., Hedayati M., Goosheh B., Bayat N. (2013). Effects of short-term aerobic exercise with and without external loading on bone metabolism and balance in postmenopausal women with osteoporosis. Rheumatol. Int..

[121] Vincent K.R., Braith R.W. (2002). Resistance exercise and bone turnover in elderly men and women. Med. Sci. Sports Exerc..

[122] Lester M.E., Urso M.L., Evans R.K., Pierce J.R., Spiering B.A., Maresh C.M., Hatfield D.L., Kraemer W.J., Nindl B.C. (2009). Influence of exercise mode and osteogenic index on bone biomarker responses during short-term physical training. Bone.

[123] Glover S.J., Eastell R., McCloskey E.V., Rogers A., Garnero P., Lowery J., Belleli R., Wright T.M., John M.R. (2009). Rapid and robust response of biochemical markers of bone formation to teriparatide therapy. Bone.

[124] Almirol E.A., Chi L.Y., Khurana B., Hurwitz S., Bluman E.M., Chiodo C., Matzkin E., Baima J., LeBoff M.S. (2016). Short-term effects of teriparatide versus placebo on bone biomarkers, structure, and fracture healing in women with lower-extremity stress fractures: A pilot study. J. Clin. Transl. Endocrinol..

[125] Liu C.T., Sahni S., Xu H., McLean R.R., Broe K.E., Hannan M.T., Boyd S.K., Bouxsein M.L., Kiel D.P., Samelson E.J. (2018). Long-Term and Recent Weight Change Are Associated With Reduced Peripheral Bone Density, Deficits in Bone Microarchitecture, and Decreased Bone Strength: The Framingham Osteoporosis Study. J. Bone Miner. Res..

[126] Inman C.L., Warren G.L., Hogan H.A., Bloomfield S.A. (1999). Mechanical loading attenuates bone loss due to immobilization and calcium deficiency. J. Appl. Physiol..

